# Credit Assignment during Movement Reinforcement Learning

**DOI:** 10.1371/journal.pone.0055352

**Published:** 2013-02-08

**Authors:** Gregory Dam, Konrad Kording, Kunlin Wei

**Affiliations:** 1 Department of Behavioral Sciences, University of Rio Grande, Rio Grande, Ohio, United States of America; 2 Department of Physical Medicine and Rehabilitation, Rehabilitation Institute of Chicago, Northwestern University, Chicago, Illinois, United States of America; 3 Department of Psychology, Key Laboratory of Machine Perception (Ministry of Education), Beijing Engineering Research Center of Intelligent Rehabilitation Engineering, Peking University, Beijing, China; The University of Western Ontario, Canada

## Abstract

We often need to learn how to move based on a single performance measure that reflects the overall success of our movements. However, movements have many properties, such as their trajectories, speeds and timing of end-points, thus the brain needs to decide which properties of movements should be improved; it needs to solve the credit assignment problem. Currently, little is known about how humans solve credit assignment problems in the context of reinforcement learning. Here we tested how human participants solve such problems during a trajectory-learning task. Without an explicitly-defined target movement, participants made hand reaches and received monetary rewards as feedback on a trial-by-trial basis. The curvature and direction of the attempted reach trajectories determined the monetary rewards received in a manner that can be manipulated experimentally. Based on the history of action-reward pairs, participants quickly solved the credit assignment problem and learned the implicit payoff function. A Bayesian credit-assignment model with built-in forgetting accurately predicts their trial-by-trial learning.

## Introduction

When a golfer decides to swing, the movement decision involves judging, among other things, the target distance, wind strength and direction, and how to handle the chosen club. The error in the resulting trajectory and the end position of the ball has many fewer dimensions than the golfer’s swing decision. In fact, most movement decisions we face have many choice dimensions that determine the final outcome. Often the feedback we obtain regarding the success of our decisions is low-dimension and may not tell how well we did with regard to every choice dimension. In such many-to-one mapping situations efficient learning should involve discovering what properties of the movement contributed to error and correcting them accordingly in order to achieve the desired outcomes.

Learning what property of a decision has resulted in an error is referred to as the *credit assignment problem* in the domains of machine learning and neurobiology [1], [2], [3]. However, little is understood about the behavioral strategies people use to solve the credit assignment problem during movement decision-making [4]. For efficient motor learning, it is necessary to discover the important movement properties and optimizing them to maximize rewards. This can be difficult due to the aforementioned many-to-one mapping between movement properties and movement outcome. More often than not, the results of movement decisions may not speak directly to each individual movement property. When a basketball player makes a jump shot, a short shot to the basket might be caused by an early release of the ball, insufficient acceleration of the wrist rotation, or lower-than-expected jump height, or a combination of all these movement properties. The challenge left to the player is to determine what properties of the movement decision should be blamed for a failure.

The many-to-one mapping between movement properties and movement outcome, traditionally referred to as the degrees of freedom problem, has been extensively investigated in motor control [5], [6]. Previous studies put emphasis on how different movement properties are coordinated and controlled to produce a desired movement goal. However, few studies have been conducted, from the perspective of reinforcement learning, on the reverse yet mathematically equivalent problem [7] of how the nervous system learns to differentially adjust different properties of a movement based on limited reward information. This type of credit assignment problem can be conveniently investigated in goal-directed movement tasks such as throwing or shooting [8], [9]. In these tasks a combination of movement properties can uniquely determine a single performance score. This opens the window for us to quantitatively vary reward functions and to examine whether and how people assign credits to different movement properties during movement reinforcement learning.

Here we investigate how people solve the credit assignment problem while learning a motor skill with reinforcement. Participants were asked to match their reaching movements to invisible target trajectories that varied in both direction and curvature. These two movement properties were differently weighted to determine the monetary reward that was presented at the end of each trial. Across two different groups of participants we varied the weighting of movement direction and curvature to the rewards. By examining the differences in learning strategies between groups we were able to investigate how participants learn to assign credit to different movement properties. We find that participants’ exploratory strategies are sensitive to the weighing of reward functions, suggesting that they are able to solve the credit assignment problem quickly with limited feedback. Furthermore participants use reward information efficiently in trial-by-trial exploration behaviors, in a manner consistent with a Bayes-like strategy.

## Methods

### The Experiment

**Figure 1:**
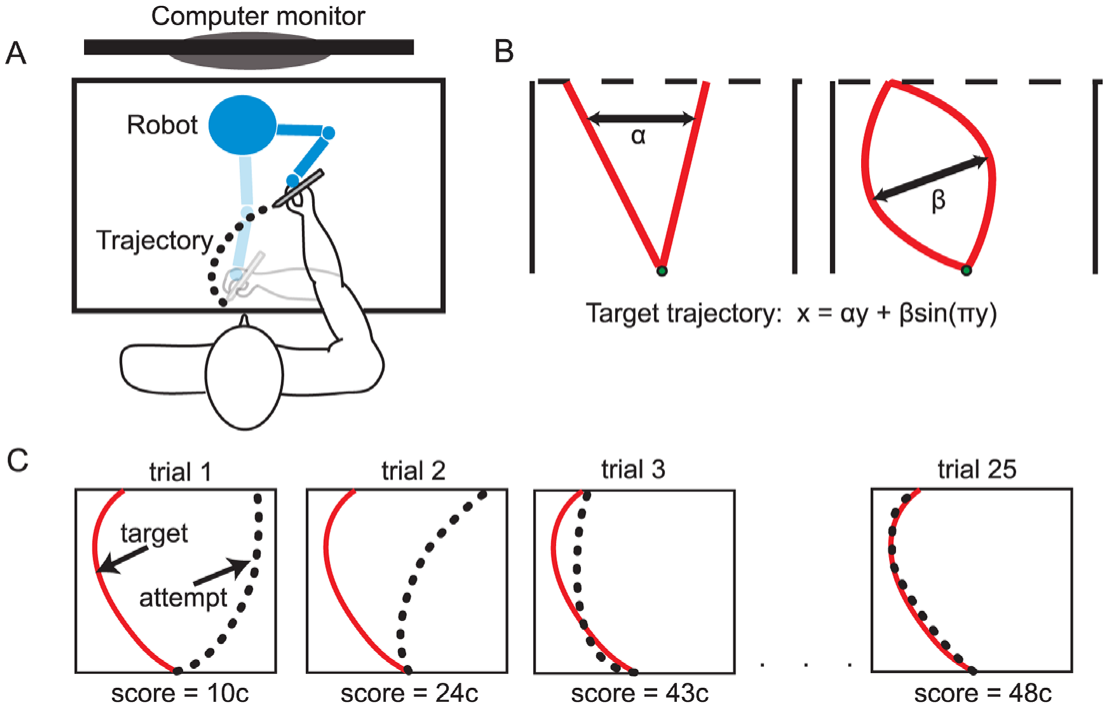
The experimental setup. A): The cartoon illustration of the setup. Participants sat before a desk and made movement trajectories on the horizontal desktop with a hand-held stylus. The hand displacement was registered by a robot. The feedback was provided via a computer monitor placed on the desk. B): A graphical representation of how trajectories were varied in both direction (α) and curvature (β). C): The learning progress of matching trajectories to a hidden target trajectory within a session of 25 trials.

Experimental techniques were approved in accordance with Northwestern University’s Policy statement on the use of humans in experiments and with federal guidelines. Informed written consent was obtained from sixteen right-handed, healthy participants (five males; mean age of 28.14 years (SD = 5.07)). Participants were seated in a chair facing a computer screen (Fig. 1A). They were asked to draw trajectories with their dominant hand using the stylus of a PHANToM Premium 1.0 haptic robot (SensAble Technologies, Inc., Woburn, MA). Trajectories were made by sliding the tip of the robot’s stylus along the surface of a desk. With the stylus, participants controlled the position of a cursor on the computer screen. Each participant attempted to match 50 different, invisible target trajectories that varied randomly in both direction and curvature. For each target trajectory participants made 25 successive attempts to approximate as closely as possible each desired target trajectory, resulting in a total of 1250 movements for the experiment. All movements started from the same position at the center of workspace, 10 cm from the table edge in front of the subject. The reaches ended when the cursor reached a displayed line 7 cm in front of (Y-direction) from the starting position. A beep was played by computer speakers to indicate the end of the trial. The shape (X and Y coordinates) of the target trajectory was defined by
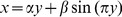
(1)where the x-coordinates (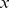) of the target trajectory are determined by two parameters, direction (α) and curvature (β). For each target trajectory, these two parameters were chosen randomly within the interval [−1, 1] with a fixed incremental size of 0.1. As a result, all target trajectories were confined in a quadrangular table-top space of 21.7 cm in width and 7 cm in depth. Note the Phantom robot’s measuring range is 25.4 cm wide and 12.7 cm deep. The stylus is also light-weighted with small inertial. Thus, neither the participants’ biomechanics, nor the mechanical constraints of the robot are likely to limit the ability to accurately match the target trajectories.

Each attempted trajectory resulted in a monetary score that provided participants with information on how closely the hidden target trajectory was matched. The score was shown on the top-left corner of a computer screen after each trial. The score had a maximum achievable amount of 50 cents. Participants were informed that they would receive the highest monetary reward they achieved from the 25 trajectory attempts for each target trajectory. Received monetary amounts were summed for all 50 target trajectories making 25$ the maximum achievable participation stipend. To determine the score, the position of the cursor was recorded at an approximate sampling frequency of 250 Hz. The participants’ attempted trajectory was then fitted to eq.1 to obtain the estimates of α and β. The magnitude of the monetary reward was determined using the difference between the estimated and the target α and β. These two errors were weighted differently for different experimental conditions.

Participants were split into two groups with eight participants in each group. One group learned target trajectories where the rewards were computed using a reward function with a large weight on the α parameter:
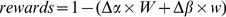
(2)where Δα and Δβ are the normalized, absolute error for direction and curvature, respectively. They will usually take the values between 0 and 1 if the actual direction and curvature are within the range of target trajectories. *W* and *w* stand for large and small weights for penalizing the direction and curvature error, respectively. They were set at 0.8 and 0.2 in this condition. The resulting 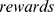 will then be multiplied by 50 cents as the actual monetary reward shown at the end of each trial. The effect of this differential weight is that that errors in the direction of attempted trajectories lead to larger reductions in obtained monetary rewards than errors in the curvature of trajectories. In rare cases the curvature and direction that participants performed with can exceed the range of target trajectories, resulting in a large error and a negative reward value. However, we only displayed a zero-reward for these trials.

The other eight participants learned target trajectories while provided with feedback using a heavily weighted β parameter of the reward function:
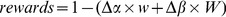
(3)Where the curvature of the attempted trajectory has a larger weight *W* and the direction has a smaller weight *w*. The two conditions associated with these two reward functions were termed α-reward and β-reward conditions, respectively.

One of the unique affordances of this experimental design is that the composite reward function above can be weighted differently for each dimension of movement (direction or curvature). By suppressing or augmenting the consequences of errors made in each movement dimension individually, we can study people’s behavioral structural credit during movement reward learning.

### The Bayesian Model

A good learning strategy would efficiently explore different movement properties while maintaining a memory of past movements and their associated rewards. Within a decision theoretic framework such a strategy can be formalized using Bayesian decision theory. The optimal decision to approximate the target trajectory combines the best *present* estimate (likelihood) with information about *past* (prior) trajectories as shown in the following equation:
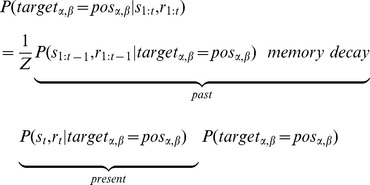
(4)


At any trial *t*, the prior is defined as the probability that the past rewards (*r_1:_*_t−1_) and attempted trajectory (*s_1:_*_t−1_) would have been observed under the assumption of a given target (*target*_α,β_ = *pos*_α,β_). The likelihood probability is defined as the probability of the current trajectory and reward (*s_t_,r_t_*) given the target shape (*target*_α,β_ = *pos*_α,β_). Furthermore, it is known that peoples’ memory of past decisions is less than perfect on such tasks [10]. To incorporate this a memory decay term (bounded in [0,1]) was added. Finally, the probability that the target trajectory (*target*_α,β_) has a given shape (*pos*_α,β_) at trial *t* is equal to probability of combining the prior, the likelihood and the memory decay.

**Figure 2:**
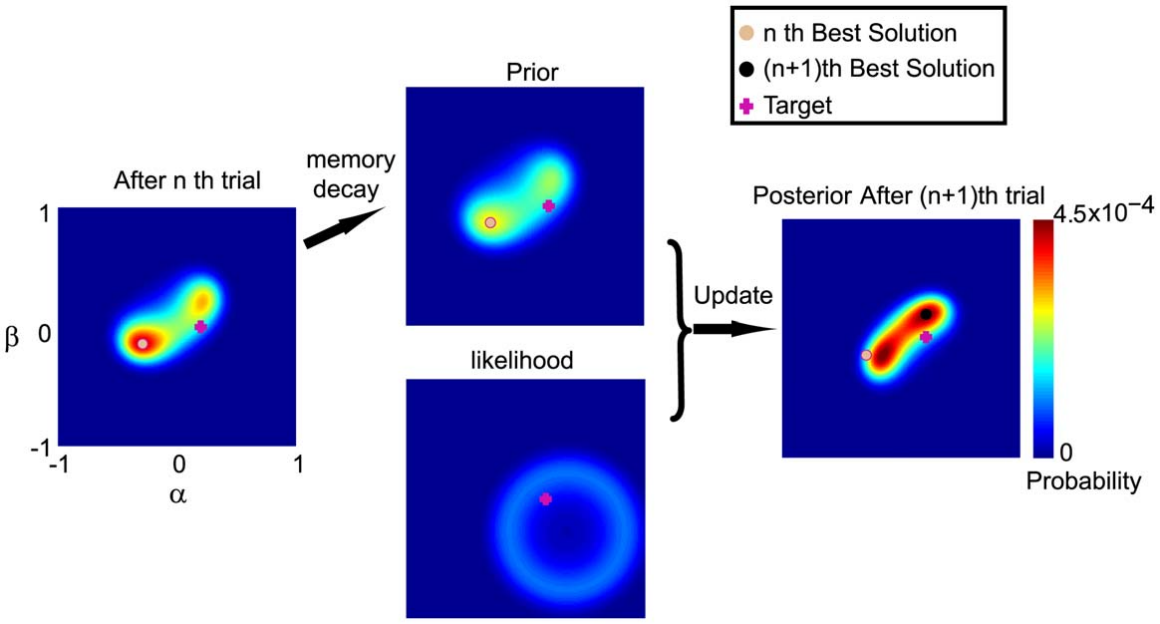
The implementation of the Bayesian model. The implementation assumes a two-dimension probability map that is updated iteratively trial by trial. It is a 200×200 matrix to code the probability of each α-β combination. Each value in the matrix is normalized such that the sum of all possibilities on the map equals 1. The pink cross denotes the current target direction and curvature. The gray dot denotes the best solution before the current trial *t* and the black dot denotes the best solution after finishing the current trial. The map from a previous trial is degraded by memory decay and it then serves as the prior before the current trial. The prediction error, the difference between the predicted reward based on the direction and curvature used in the current trial and the actual received reward, serves as likelihood distribution to update the probability map. By combining the prior and the likelihood, the probability map is updated to form the posterior distribution. The learning is demonstrated in that the best solution of the posterior, compared to that of the prior, becomes closer to the target solution. The data are from a typical trial (the 4^th^ trial in a 25 learning sequence) from a single participant.

For implementing Bayesian updating, we assume participants maintain a probability map of the to-be-learned curvature and direction, and iteratively update that map after receiving the reward upon each trial (Fig. 2). The map codes the probability of each curvature-direction combination and decays linearly in between trials as captured by the memory decay term λ:
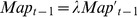
(5)


Where 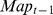 and 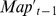 are the probability maps updated at the previous trial and before the current trial, respectively. We further assume that participants implicitly approximate the reward function by giving weights to errors in curvature and in direction. Hence, the predicted reward, 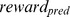, is computed as



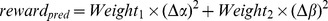
Where 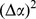 and 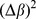 are the squared errors in both dimensions on a trial. This predicted reward is compared with the actual reward achieved on that trial (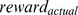) and the discrepancy (prediction error) is then used to update the probability map with a constant learning rate 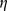:



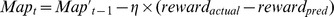
Where 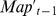 and 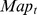 are the probability map before and after the current trial, respectively. Thus the model has 4 free parameters: the memory decay term 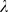, weights for errors 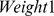 and 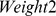, and the learning constant 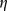. They were estimated by fitting the model to all 50 learning sequences for each participant.

## Results

Here we designed a reinforcement learning task requiring the actor to assign varying credits to different movement properties based on single monetary rewards. By assessing trial-by-trial exploration behavior, we can examine whether participants can solve this credit assignment problem efficiently and whether reward-movement pairings are sufficient to support this learning.

**Figure 3:**
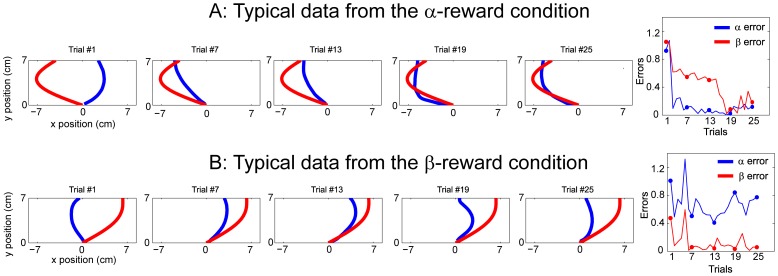
Learning data from typical participants from the α-reward condition (upper panels, A) and the β-reward condition (lower panels, B). A): Five individual trial trajectories (blue) along with its corresponding hidden target trajectory (red) are shown in separate panels from left to right. The rightmost panel displays the absolute error in trajectory direction (α) and curvature (β). The direction of targets is learned earlier than curvature. B): Panels follow the same format as the upper panels. Curvature learning occurs early but the errors in the direction of targets remain high throughout the session.

We first examined whether participants could learn the hidden target trajectory with monetary rewards as feedback (Fig. 3). Data from a typical participant in the α-reward condition indicates that the movement trajectories incrementally match the hidden target trajectory in terms of the direction and the curvature (Fig. 3A). For this particular participant, the learning of two trajectory properties reaches a plateau within 25 trials. However, not all the target trajectories are learned equally well. A typical participant from the β-reward condition exhibits improvement in trajectory curvature early on but fails to minimize direction error within 25 trials (Fig. 3B).

**Figure 4:**
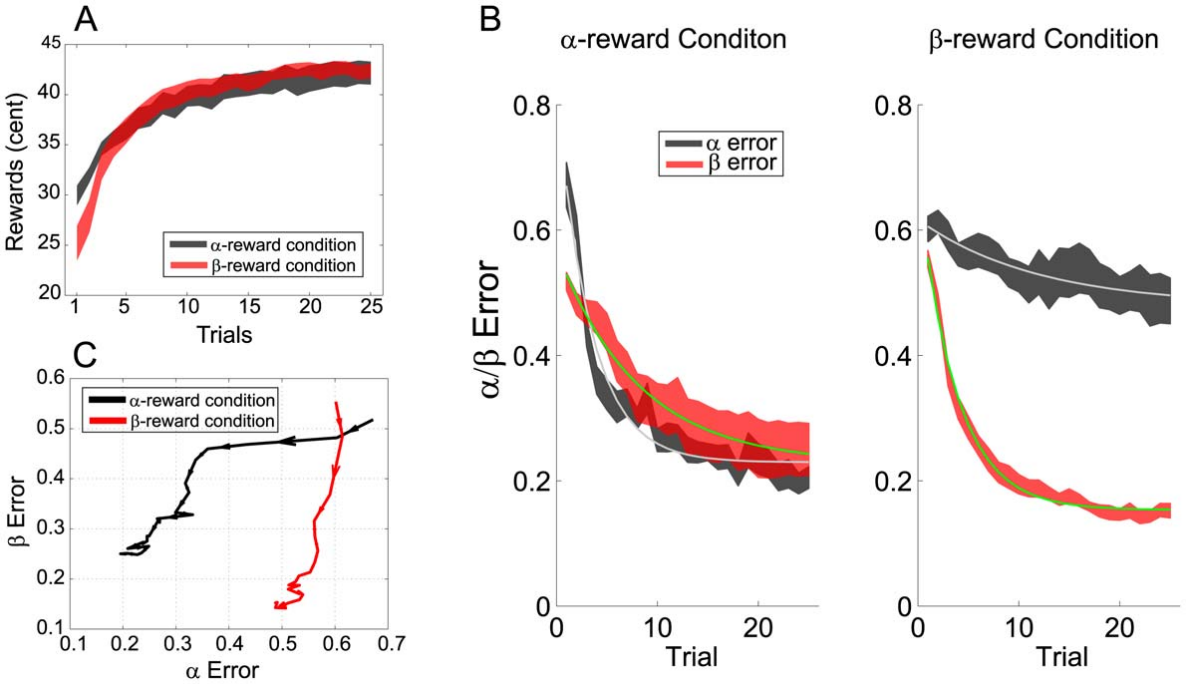
Learning-related changes. A): The monetary rewards, averaged over all target trajectories, are plotted as a function of trials. The two shaded curves stand for mean±SEM over participants for two reward conditions separately. B): The learning curves of two trajectory properties, direction (α) and curvature (β), are plotted in black and red, respectively. The green and the gray lines denote their corresponding exponential fits. The results from the two reward conditions are presented in two separate subplots. C): The same learning curves from B) are re-plotted in the α-β space. The arrows indicate directions of changes and their sizes are proportional to magnitude of changes.

A group analysis reveals a similar picture. For both reward conditions, participants exhibit exponential learning and their performance (in terms of rewards received) asymptotes around the 20^th^ trial (Fig. 4A). The average highest monetary reward achieved across participants and across all 50 target trajectories is 45.6 cents (SD = 3.8), which is very close to the maximum possible reward of 50 cents. Participants demonstrate better initial performance in the α-reward condition, perhaps suggesting that the reach direction is inherently easier to learn than its curvature. The learning curves of the reward from the two conditions are similar but those of two movement properties are different (see below). Overall, these results suggest that participants are very adept at learning new target trajectories in 25 attempts.

If one of the movement properties was weighted more in the rewards, we would expect that participants should preferentially explore that property. This is indeed the case (Fig. 4B). The error in the weighted property is corrected more rapidly. By fitting an exponential function to the average learning curve of each property, we find that the decay constant in the α-reward condition is 3.3 and 8.1 for α and β, respectively. In contrast, in the β-reward condition it is 11.8 and 3.7 for α and β, respectively. These results indicate that the weighted movement property is learned faster than the un-weighted one. Participants also settle at different asymptotes after 25-trial learning in two conditions: for the α-reward condition both α and β errors settles at 0.23, while for the β-reward condition α error settles at 0.48 and β error at 0.15. In the β-reward condition, α error remains high late into the learning sequence even though the reward reaches a plateau. In sum, the learning rate of the weighted property is substantially faster than that of the less-weighted despite that learning of the two properties is completed to different extents.

The preferential improvement in the weighted property can be further demonstrated by casting the two learning curves into a two-error-dimension space (Fig. 4C). Learning happens predominantly in direction of the trajectory when the reward function is α (direction) weighted and predominantly in curvature when the reward function is β (curvature) weighted. These results suggest that participants are able to infer the most important dimension in the movement learning task based on a feedback history of uni-dimensional monetary rewards.

**Figure 5:**
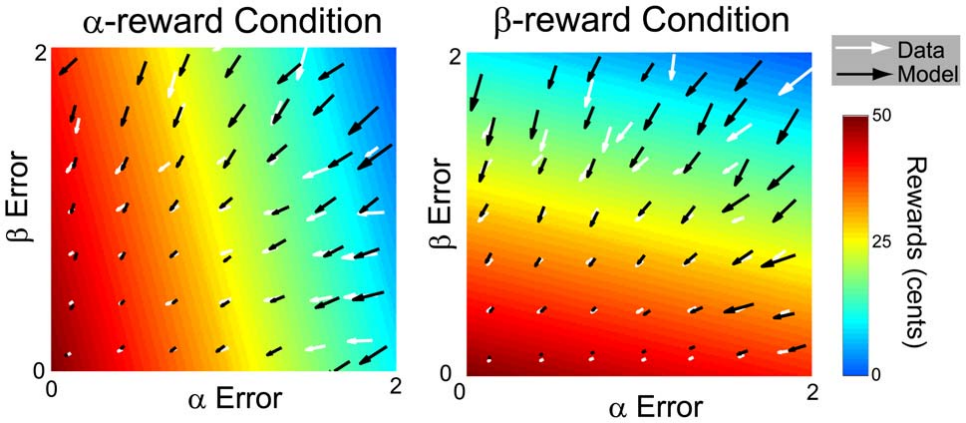
Quiver plot of average changes in movement properties with a α- (A) and a β- (B) reward function. The x- and y-axis denote the absolute errors in two movement properties, respectively. The background temperature plots display the reward functions with the maximum achievable reward centered at zero alpha-beta error. The higher the temperature the higher the reward. White vectors are average parameter changes from one trial to the next across all target trajectories and all participants. Black vectors represent the Bayesian model’s predictions.

In order to understand how participants used information about trajectory and rewards, a Bayesian model was developed and fitted to the participants’ movement data. The average changes in trajectory properties between subsequent attempts are shown in a quiver plot (Fig. 5). Vectors are sparse as only a limited set of possible transitions are observed among our participants. Those long vectors, indicating large corrections over successive trials, mostly appear when large curvature and/or direction errors occur. Some trials resulted in small or even negative rewards but they constitute only a small fraction of total trials (1.19% and 0.09% for the α- and β-reward conditions, respectively). Note that zero is the lowest reward feedback given on any trials. Overall, the Bayesian model captures the trial-by-trial changes in trajectory properties, shown by the close match between data and model vectors in the terms of direction and magnitude. The Bayesian model explains 42.9% and 49.6% of total variance in participants’ trajectories decisions for the α- and β-reward conditions, respectively. This suggests that participants might employ a near optimal strategy for updating information about trajectory shape and rewards, and use this knowledge to inform subsequent trajectory decisions. Interestingly, the memory decay term is 0.32±0.1 and 0.54±0.1 for α- and β- reward conditions, respectively. This suggests that participants relied on only a fraction of past information to guide the current search.

**Figure 6:**
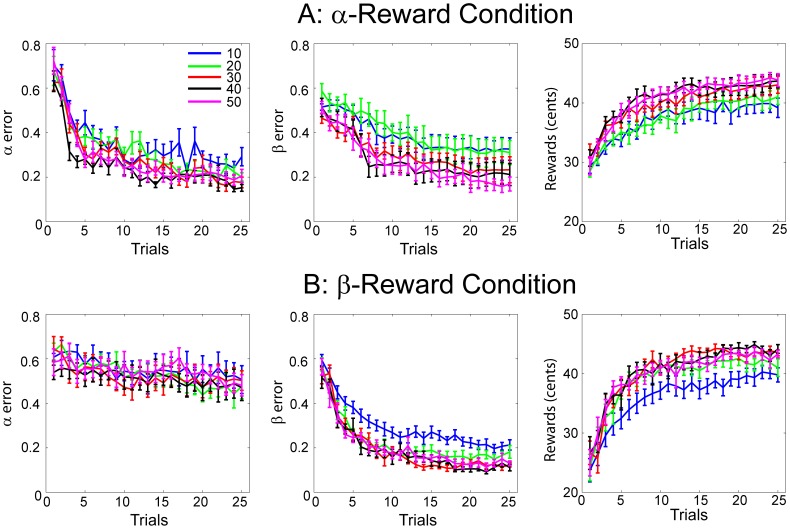
Average learning curves over each of 10 target trajectories are shown for the α-reward condition (A) and the β-reward condition (B) separately. Displayed in different panels, learning curves are based on α error, β error and actual monetary rewards.

Is it possible that the ability of matching invisible targets was improved gradually over the course of experiment? We test this possible meta-learning effect by evaluating learning curves for different parts of the experiments. The total 50 target trajectories are grouped into 5 sessions, 10 targets each. The learning curves, based on α error, β error and rewards are shown separately (Fig. 6). We quantified the amount of learning by taking the average of the last 5 trials and compared them across sessions. For the α-reward condition, there is no significantly difference in time for all three variables despite an overall improving trend during the course of experiment. For the β-reward condition, the amount of learning is significantly larger in terms of β error and rewards between the first session and the last sessions (post-hoc Tukey tests, *p*<0.05). Taken together, a meta-learning effect is present but it is confined to the initial learning stages during the β-reward condition only.

## Discussion

We have examined how people learn to move to match invisible target trajectories with only single monetary rewards as feedback after each movement. We show that participants solve this credit assignment problem quickly and converge to the correct solution despite the many-to-one mapping of the learning task. Their search for the target is systematically affected by the properties of the reward function used as demonstrated by faster learning of the more rewarded movement property. Participants learn quickly what movement properties matter most. Their trial-by-trial learning is consistent with the predictions of a Bayesian model, suggesting that they efficiently use reinforcement information during learning.

Even though participants quickly and successfully discovered the heavily-weighted error dimension, the two movement properties were not learned equally. Specifically, in the β-reward condition participants failed to optimize the direction error to the same degree as that of the curvature error, even though they were apparently capable of doing so as shown in the α-reward condition (Fig. 4). At the same time, despite of this relatively large direction error asymptote, similarly good rewards were achieved by optimizing the curvature. Thus, this large asymptote might reflect that importance of reward feedback [11]: participants are slow in optimizing less-penalized dimension when reward feedback is already improved to a plateau. It is interesting that in the α-reward condition the curvature error, the less-penalized dimension, did not exhibit similarly slack learning. This difference thus highlights that less-penalized movement properties are not optimized equally fast when only overall reward feedback is provided. This is interesting since when the same two movement properties are heavily-weighted their learning was equally fast. The system is thus efficient in terms of preferentially dealing with more important control variables as the minimum-intervention principle suggests [6].

Our findings show that search behavior in reinforcement motor learning is shaped by the properties of the reward function as the more rewarding dimension is preferentially learned. Analogous findings has been demonstrated when analyzing the control of movements where a reduction in variability of the more performance-affected dimension is observed [9], [12], [13], [14], [15]. In these studies, the relationship between control variables (e.g., joint angles) and a performance measure (e.g., the endpoint precision of a reach movement) have been characterized by certain analytic functions termed solution manifolds. Often times, the interactions between various control variables are of particular importance for good performance, as prescribed by the structure of a solution manifold. A universal finding in these studies is that people control their movements so as to minimize the variability of the critical dimensions as such inherent motor variability will minimally impact performance {Martin, 2002 #1303;Kudo, 2000 #1304}. These observed covariations between control variables reflect the established synergy [18] or strategies based on optimal feedback control [6]. Conceptually, the solution manifold is equivalent to the reward function in the present study and both of them map movement properties to a performance measure. Our findings thus suggest that *before* a stable performance strategy is formed the nervous system *learns* to first modify the movement properties that matter the most for movement performance. This preferential error reduction is flexible and dynamically adjusted according to task demands that are experimentally varied with the use of different reward functions.

Some effort has been devoted towards studying the *temporal* credit assignment problem in reinforcement learning of motor skill. In these tasks, an agent needs to judge *when* was an action that resulted in error committed during the execution of a sequence of actions [19]. Accordingly, these studies use action sequence learning tasks such as maze-searching {Tolman, 1930 #1403;Fu, 2006 #1404}, sequential choice task {Fu, 2008 #1402} and mine-field navigation {Gordon, 1994 #1401}. The tasks are designed to test how credits are assigned to actions in multistep choice situations. Our study instead addresses the *structural* credit assignment problem [24], [25], focusing on how the agent assigns credits to different properties of a single action. The agent needs to learn *what* property of a decision lead to error in order to correct it, an important problem that has received little attention. This work represents an initial endeavor to study the structural credit assignment problem within the domain of motor learning.

The close match between participants’ movement data and the predictions of a Bayesian model suggests that people efficiently integrate past and present information to inform subsequent movement decisions. Our Bayesian model is conceptually similar to other reinforcement learning models where the decision for the next attempt is made on the basis of the history of reward-movement pairs. An interesting finding is that the memory decay between successive attempts was fairly high (0.32 and 0.54, where 1 represents perfect recall). Trial-by-trial search behavior has a heavy temporal discounting component and past action-reward pairs have limited influence on the current decision. This suggest that either people have poor recall of past action-reward parings, or that they are over-confident of their current exploratory strategies, neglecting information gained from previous exploration.

We acknowledge that our model is simplified by assuming constant memory decay and learning rate over the whole learning process. The assumption made is that learning is essentially the same over the course of the experiment. Our results indicate that there is some evidence of meta-learning in the β-reward condition where the learning is improved after exposure to 10 target trajectories. Future modeling efforts could incorporate small meta-learning effects to capture the data better. On the other hand, to our best knowledge there is no good alternative model for capturing the trial-to-trial learning for this type of structural credit assignment task even though similar tasks have been investigated in motor learning {Müller, 2004 #892;Scholz, 1999 #1300}.

Credit assignment problems also arise in motor adaptation where the nervous system adapts movement to control perturbations from external environment or from noise in the movement system itself. It has been proposed that adaptation depends on probabilistic inference of causality of these changes: movement perturbations that are less likely to be caused by changes in motor apparatus are less likely to be learned and generalized [3]. The movement errors that are less likely caused by the actor and therefore more likely caused by external perturbations are also less likely to be corrected [26]. These studies suggest that the nervous system assigns different weights to movement error or perturbation according to the inferred causal structure of error source during motor adaptation. In the present study, all errors are attributed to the actor who is able to efficiently learn what properties matter most with regards to the desired goal. In the reinforcement task used in our experiment, no single action-reward pair could reveal the structure of the payoff function. Remarkably, participants are able to quickly glean the pay-off function by exploring different movement properties and noticing how they affect the magnitude of rewards. The movement property that has the greatest effect on performance is learned faster and the credit assignment problem is solved efficiently.
